# Peripheral CD4^+^ T Cell Cytokine Responses Following Human Challenge and Re-Challenge with *Campylobacter jejuni*

**DOI:** 10.1371/journal.pone.0112513

**Published:** 2014-11-14

**Authors:** Kelly A. Fimlaid, Janet C. Lindow, David R. Tribble, Janice Y. Bunn, Alexander C. Maue, Beth D. Kirkpatrick

**Affiliations:** 1 Department of Microbiology and Molecular Genetics, University of Vermont, Burlington, Vermont, 05405, United States of America; 2 University of Vermont College of Medicine, Vaccine Testing Center and Unit of Infectious Diseases, Burlington, Vermont, United States of America; 3 Infectious Disease Clinical Research Program, Uniformed Services University of the Health Sciences, Bethesda, Maryland, United States of America; 4 University of Vermont College of Mathematics, Burlington, Vermont, United States of America; 5 Naval Medical Research Center, Enteric Diseases Department, Silver Spring, Maryland, United States of America; Charité-University Medicine Berlin, Germany

## Abstract

*Campylobacter jejuni* is a leading cause of human gastroenteritis worldwide; however, our understanding of the human immune response to *C. jejuni* infection is limited. A previous human challenge model has shown that *C. jejuni* elicits IFNγ production by peripheral blood mononuclear cells, a response associated with protection from clinical disease following re-infection. In this study, we investigate T lymphocyte profiles associated with campylobacteriosis using specimens from a new human challenge model in which *C. jejuni*-naïve subjects were challenged and re-challenged with *C. jejuni* CG8421. Multiparameter flow cytometry was used to investigate T lymphocytes as a source of cytokines, including IFNγ, and to identify cytokine patterns associated with either campylobacteriosis or protection from disease. Unexpectedly, all but one subject evaluated re-experienced campylobacteriosis after re-challenge. We show that CD4^+^ T cells make IFNγ and other pro-inflammatory cytokines in response to infection; however, multifunctional cytokine response patterns were not found. Cytokine production from peripheral CD4^+^ T cells was not enhanced following re-challenge, which may suggest deletion or tolerance. Evaluation of alternative paradigms or models is needed to better understand the immune components of protection from campylobacteriosis.

## Introduction

*Campylobacter jejuni* is among the most common enteric bacterial pathogens causing gastrointestinal disease. On a global scale, approximately 400-500 million people experience campylobacteriosis annually [1]. Ingestion of *C*. *jejuni*-contaminated food or water can cause a spectrum of disease symptoms, which include diarrhea, fever, and abdominal cramping [2], [3]. While infection is self-limited in most healthy individuals, antibiotic therapy is necessary in severe cases and in immunocompromised individuals. A major concern associated with campylobacteriosis is the potential for post-infectious sequelae including the demyelinating neurologic disease, Guillain-Barré syndrome, reactive arthritis, and post-infectious irritable bowel syndrome [4], [5]. Furthermore, increasing emergence of antibiotic resistant strains highlights the importance of the development of new therapeutics and prevention strategies which require a better understanding of the human immune response to *C. jejuni* infection [6].

Characterization of human immune responses that contribute to protection from clinical illness caused by *C. jejuni* has proven challenging. Information gathered from natural *C. jejuni* infection can only be cautiously interpreted, since inoculum and time from exposure is not known. Protection from clinical disease caused by *C. jejuni* also appears to vary with age, strain, and exposure history [7]–[10]. Further, the absence of a small animal model that shares characteristics of human disease has made mechanistic studies of the immune response to *C. jejuni* infection extremely difficult [11]–[14]. While important advances have been made, the human immune response to *C. jejuni* infection has not been fully characterized and more studies are needed to determine the immunologic responses that develop as a result of disease for vaccine design and drug development.

In human disease, the role of CD4^+^ T cells in adaptive immune responses to *C. jejuni* infection has not been characterized. Human *C. jejuni* experimental infection or ‘challenge’ models provide a unique opportunity to evaluate these immune responses. Analysis from a previously performed human challenge and re-challenge model using *C. jejuni* strain 81-176 showed association between pre-infection levels of IFNγ and protection from clinical campylobacteriosis [13]. Cellular immune responses in other infection models have also been investigated. For example, human colonic explants infected with *C. jejuni* exhibited marked increases in IFNγ production following infection [14]. Additionally, in a *C. jejuni*-resistant C57BL/6 mouse model, *C. jejuni*-infected dendritic cells resulted in CD4^+^ T_H_1 polarization and IFNγ production [15].

In the present study, we used multiparameter flow cytometry to evaluate the T cell cytokine profiles from individuals infected with *C. jejuni* CG8421 in an experimental challenge and re-challenge model [12]. We sought to confirm that CD4^+^ human T cells from *C. jejuni*-infected subjects produce IFNγ, following *ex vivo* stimulation. We asked whether these T cell responses were multifunctional (capable of producing multiple cytokines simultaneously) since multifunctional T cells have been associated with long-term immunity and protection from disease progression for a variety of bacterial, viral, and parasitic pathogens [16]–[18]. Our data demonstrate a consistent pattern of pro-inflammatory cytokine production by *C. jejuni*-specific CD4^+^ T cells following primary infection, which included IFNγ, TNF-α, IL-2, and the chemokine, MIP-1β. Interestingly, upon re-infection, there was no enhancement of the CD4^+^ cytokine response in any subject. Low quantities of multifunctional T cell responses were observed post infection, however a clear kinetic pattern was not distinguishable. One subject evaluated in this re-infection model was protected from clinical illness upon re-infection, and interestingly, this subject shared similar CD4^+^ T cell profiles to study participants that were not protected from re-infection. Overall, this is the first detailed description of the human CD4^+^ T cell response to primary and secondary *C. jejuni* infection and offers novel insights into the complexity of immune protection from campylobacteriosis.

## Materials and Methods

### *C. jejuni* CG8421 experimental infection trials

Peripheral blood mononuclear cells (PBMCs) used in this analysis were collected under two separate *C. jejuni* CG8421 inpatient trials as previously described [12]. Of note, volunteers were excluded if they had clinical or immunologic evidence (IgA or IFNγ production) of prior exposure to *C. jejuni*. Written informed consent was obtained from all subjects. Clinical protocols were approved by the Institutional Review Boards at University of Vermont and the Naval Medical Research Center. Clinical trials are registered as *Campylobacter jejuni* Challenge Model Development: Dose Ranging Study NCT00434798 (Trial 1) and *Campylobacter jejuni* Challenge Model Development: Assessment of Homologous Protection NCT01048112 (Trial 2).

### PBMC collection and handling

Blood samples were collected in EDTA tubes; PBMCs were isolated using AccuSpin tubes within 4 hours of collection. Cells were cryopreserved in freezing media (Sigma) and were thawed in 37°C complete media [cRPMI-10FCS: RPMI-1640 (GIBCO), 10% fetal calf serum (HyClone), 1% penicillin/streptomycin (Sigma), 2 mM L-glutamine (GIBCO)], and 2.72 units DNase/mL media (NEB). Cells were pelleted (300*xg*, 10 min), and washed with cRPMI-10HS [RPMI-1640, 10% human serum (Gemini Bio-Products), 50 µM β-mercaptoethanol (Sigma), 2 mM L-glutamine, and 50 µg/mL gentamicin (GIBCO)]. Cells were resuspended in cRPMI-10HS and rested 12 hours at 5–10×10^6^ cells/mL. PBMCs were washed once with cRPMI-10HS, reconstituted to 1–1.5×10^7^ cells/mL, and 100 µl was aliquoted into a 96-well tray well for each condition.

### *Campylobacter jejuni* antigen preparation

*C. jejuni* antigen (CAg) used to stimulate PBMCs was prepared as follows: *C. jejuni* strain CG8421 was cultured under conditions used for human challenge [8], fixed with 4% formaldehyde, washed 3 times with PBS (10,000*xg* for 5 min, at 4°C), resuspended in 1 ml PBS, and sonicated on ice using a Fisher Scientific Sonic Dismembrator Model 100 (three 20 sec intervals, 100% power). Antigen was titrated and time courses were performed to optimize PBMC stimulation conditions utilizing PBMCs from individuals with confirmed natural exposure to *C. jejuni* or subjects from previous trials (data not shown).

### *Ex vivo* PBMC stimulation

For all subjects and timepoints, PBMCs were assayed with the following negative control and stimulation conditions: i) negative control (PBS), ii) positive control [Staphylococcal enterotoxin B (SEB)], and iii) *C. jejuni* antigen (CAg). Each condition was incubated in cRPMI-10HS containing 1 µg/mL anti-CD28 and 1 µg/mL anti-CD49d for 24 hours at 37°C+5% CO_2_. Brefeldin A (10 µg/mL) was added for the final 12 hours of incubation. Subject A from Trial 1, was evaluated at time-points: D0 (*C. jejuni* naïve sample), D28, D150 (day of re-challenge), D178, and D540. The 8 subjects from Trial 2 were evaluated on D0, D7, D14, D28, D98 (day of re-challenge), D105, D112, and D126 unless specified. Unless limited by PBMCs, technical replicates of the CAg condition were performed. All PBMCs evaluated were capable of producing IFNγ based on the positive control.

### Staining procedure

After stimulation, 2 mM EDTA (final concentration) was added to each well for 10 min at room temperature (RT). A LIVE/DEAD Fixable Dead Cell Stain (blue fluorescent reactive dye; Invitrogen) was used to identify and exclude non-viable cells from the analysis. Cells were fixed (BD Lyse Solution) and permeabilized (BD Perm 2), according to manufacturer's instructions. Surface and intracellular staining were done simultaneously for 30 min at RT with the following pre-titered antibody panel: anti–CD3 PerCP Cy5.5 (UCHT1; BD), anti–CD8 V450 (RPA-T8; BD), anti–CD4 V500 (RPA-T4; BD), anti–IFNγ PE-Cy7 (B27; BD), anti–IL-2 PE (5344.111; BD), anti–TNFα FITC (6401.1111;BD), and anti–MIP-1β APC (D21-1351; BD). Cells were acquired using an LSRII flow cytometer (BD) within 24 hours and ≥200,000 total events were collected for each sample unless otherwise specified.

### Flow Cytometry Analysis

FlowJo 9.3.1 software (Tree Star, Inc.) and SPICE V5.22β software were used for flow cytometry analysis [19]. PBMCs were analyzed as follows: singlets, lymphocytes, live/CD3^+^ cells, CD4^+^ and CD8^+^ cell subsets. From the CD4^+^ and CD8^+^ cell subsets, cytokines were individually gated based on fluorescence minus one (FMO) data. GraphPad PRISM V5.0d was used for cytokine kinetic analyses. Boolean gating strategy and SPICE V5.22β were used for analysis of multifunctional cell subsets. All MFI values reported represent the median fluorescence intensity.

### Statistics

T cell cytokines were analyzed and reported as the percent of cytokines produced in the antigen stimulation condition (CAg) minus the background (negative control). Single-group repeated measures analysis of variance based on the ranks of all values was used to examine cytokines or multifunctional T cells produced at each time point (the repeating factor) relative to a pre-challenge time-point [20]. Any statistically significant results were followed by post-hoc tests to examine day-specific differences from the pre-challenge time-point. Repeated measures analyses were performed using SAS, version 9.2; Wilcoxon rank sum tests were computed using SPICE v.5.22β. P values≤0.05 were considered significant.

## Results

### Clinical Subjects

#### Subjects B-I

Eight participants in an inpatient challenge model (Trial 2) (Subjects B-I) with no clinical or immunologic evidence of prior exposure to *C. jejuni* were evaluated for T cell responses associated with infection [21]. All eight subjects received an initial inoculum of 8.6×10^5^ colony-forming units (CFU) of *C. jejuni* CG8421 and all experienced campylobacteriosis, as previously reported [21]. Following the same procedures, Subjects B-I underwent homologous rechallenge with the same strain 90 days after the initial inoculum, at a dose of 3.6×10^5^ CFU. Subjects B-I experienced campylobacteriosis following re-challenge. As reported elsewhere, protection from clinical disease following first exposure was not observed nor was there a difference in the severity of clinical illness during Trial 2 after the first and second infections [21].

#### Subject A

One subject (Subject A), part of a separate trial (Trial 1), with no clinical or immunologic evidence of prior exposure to *C. jejuni*, underwent initial challenge with 1×10^5^ CFU of *C. jejuni* CG8421 and homologous rechallenge 150 days later with the same strain dosed at a dose of 5×10^4^ CFU [12]. While Trial 1 consisted of a large cohort of people, only Subject A underwent homologous re-challenge; Subject A did not experience diarrhea or any clinical signs or symtoms consistent with campylobacteriosis upon re-challenge.

### Peripheral CD4^+^ and CD8^+^ T cells produce pro-inflammatory cytokines following primary challenge with *C. jejuni* CG8421 in naïve subjects

**Figure 1:**
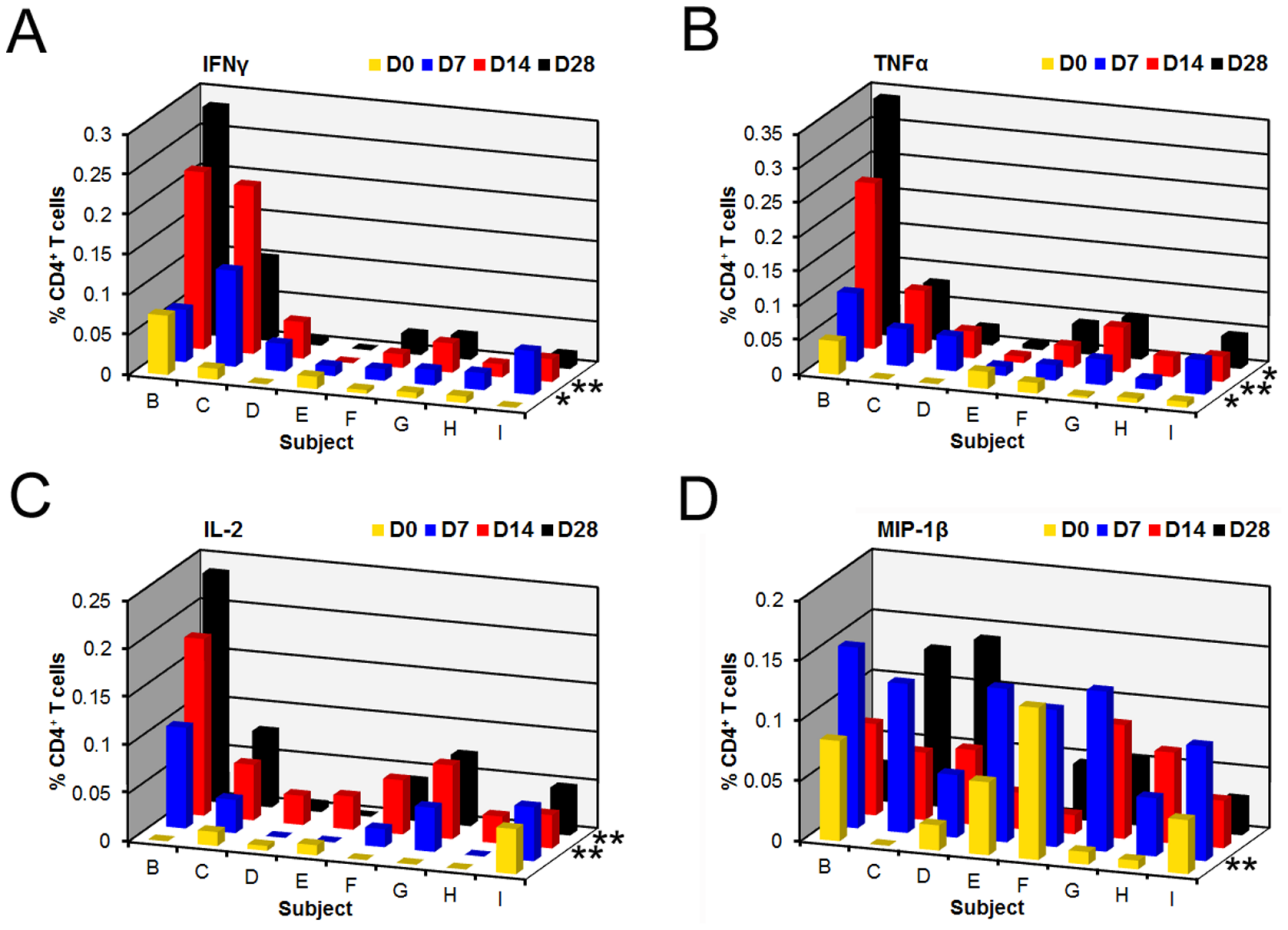
Proinflammatory cytokines and a chemokine are produced with specific kinetics post-primary infection with *C. jejuni*. We analyzed CD4^+^ T cells for the production of IFNγ^+^, TNFα^+^, IL-2^+^, and MIP-1β^+^ for eight subjects challenged with *C. jejuni* at multiple timepoints pre- and post-primary infection. Responses shown are the percentage of cytokine positive CD4^+^ T cells from CAg-stimulated PBMCs with the background percentage of cytokine-positive T cells in the negative control (PBS) subtracted. CD4^+^ T cells producing each cytokine were reported for 3 time-points post-challenge: D7 (blue), D14 (red), and D28 (black). A) CD4^+^IFNγ^+^ T cells; B) CD4^+^TNFα^+^ T cells; C) CD4^+^IL-2^+^ T cells; and D) CD4^+^MIP-1β^+^ T cells. An asterisk denotes a significant difference between a response on a post-challenge day and D0 (yellow): * P<.05 and ** P<.01. Statistics were determined using repeated measures analysis of variance were not available for Subject B after re-infection.

A representative flow cytometry gating scheme showing *C. jejuni* CG8421 antigen (CAg) compared to the negative control is shown in **Fig. S1**. PBMCs isolated from Subjects B-I were analyzed using the same *C. jejuni ex vivo* stimulation conditions, at pre- and post-primary infection time-points. Statistically significant increases in total percentages of CD4^+^IFNγ^+^, CD4^+^IL-2^+^, and CD4^+^TNFα^+^ T cells were detected post-infection relative to D0 (P<0.05, 0.01, and 0.05, respectively) following primary infection (**Fig. 1**). CD4^+^MIP-1β^+^ cells trended toward significance (P = 0.06) post-infection relative to pre-infection. Analysis of individual post-primary challenge days showed statistically significant increases in CD4^+^IFNγ^+^ cells on D7 and D14 relative to D0 (P<0.05 and P<0.01, respectively) (**Fig. 1A**). CD4^+^TNFα^+^ T cells were produced with similar kinetics as CD4^+^IFNγ^+^ T cells, exhibiting significant increases on D7, D14, and D28 relative to D0 (P<0.05 for all timepoints) (**Fig. 1B**). While CD4^+^IL-2^+^ T cell kinetics were delayed relative to those seen for CD4^+^IFNγ^+^ and CD4^+^TNFα^+^, significant levels were observed on D14 and D28 (P<0.01 and 0.01) (**Fig. 1C**). Lastly, CD4^+^MIP-1β^+^ cells were elevated at D7 compared to D0 (P<0.01) (**Fig. 1D**).

Analysis of CD8^+^ T cells showed modest increases in CD8^+^IFNγ^+^ and CD8^+^MIP-1β^+^ T cells at various time-points by subjects post initial infection, but no pattern was evident post initial infection (**Fig. S2A and S2B**). Following re-challenge, there was no clear pattern of CD8^+^IFNγ^+^ and CD8^+^MIP-1β^+^ T cell profiles (**Fig. S2C and S2D**).

### An enhanced CD4^+^ cytokine response is not observed following *C. jejuni* CG8421 re-challenge

**Figure 2:**
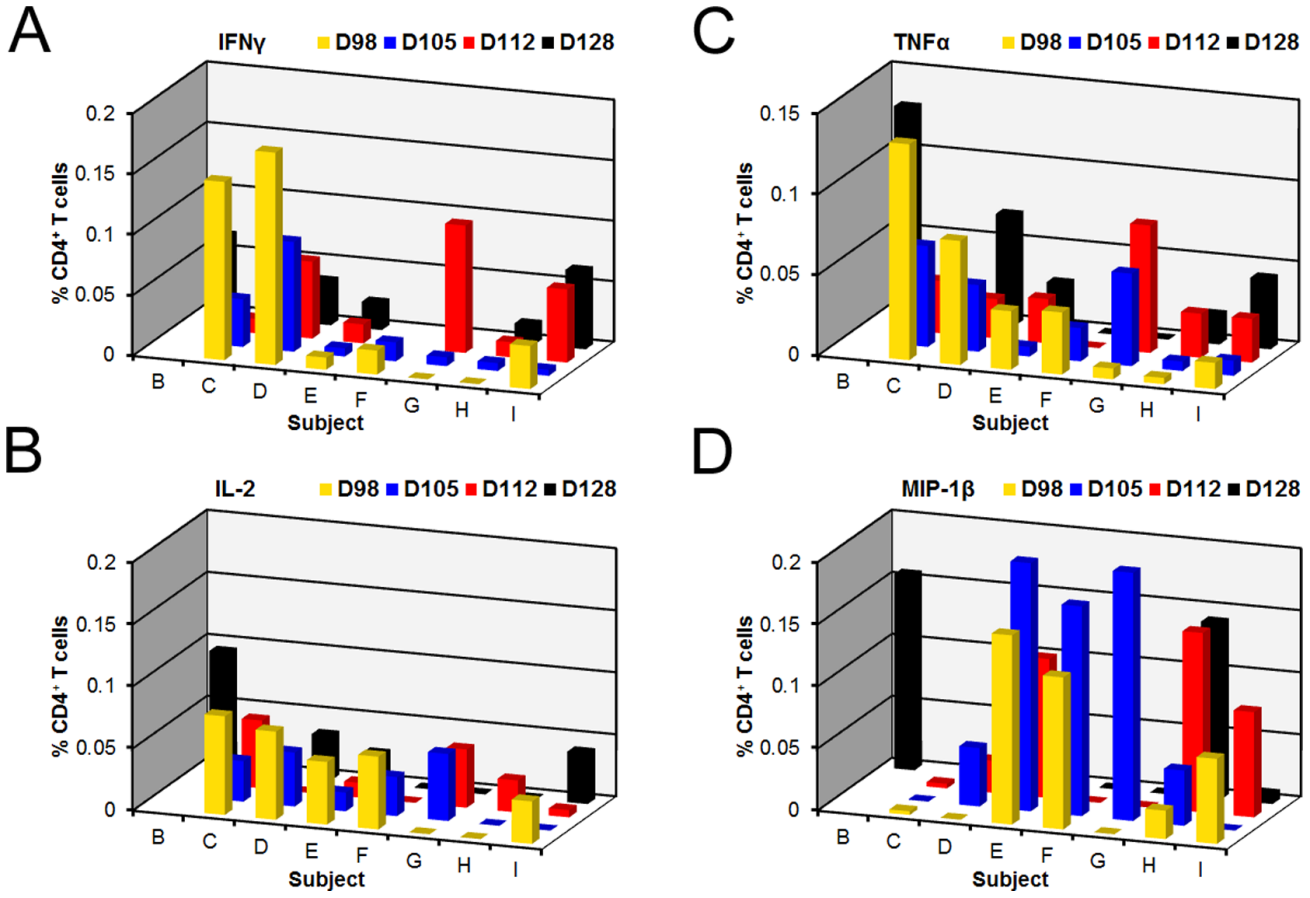
CD4^+^ T cell responses did not show a specific signature following re-challenge with the homologous *C. jejuni* strain. The effect of challenge on *C. jejuni* veterans shows a diverse T cell profile compared to naïve subjects. Eight subjects were re-dosed with the homologous *C. jejuni* CG8421 strain three months after initial infection. We examined CD4^+^ T cell responses for the production of IFNγ^+^, TNFα^+^, IL-2^+^, and MIP-1β at multiple timepoints pre- and post-re-infection. Responses shown are the percentage of cytokine positive CD4^+^ T cells from CAg-stimulated PBMCs with the background percentage of cytokine-positive T cells in the negative control (PBS) subtracted. D98 represents PBMCs from a blood drawn prior to re-infection. Bars represent percent of CD4^+^ T cells detected on specific days: D98 (day of re-dosing, yellow); D105 (blue); D112 (red); and D126 (black). No significant changes were observed post re-challenge compared to D98.

To evaluate whether an enhanced T cell response occurred following homologous re-infection, suggestive of activation of a memory T cell response, we analyzed responses following re-exposure to *C. jejuni*. Analysis of the cytokine kinetics post re-exposure in subjects that did not have protection from clinical disease showed variable responses compared to what was seen after first exposure (**Fig. 2A-D**); PBMCs were not available for all subjects on all days during the re-challenge denoted by a missing square from the figure plane. Overall, the cytokine responses at time-points following re-challenge were not significantly different relative to day of re-challenge (D98) or D0 for any of the cytokines under investigation.

### Mono-functional CD4^+^ T cells are the dominant T cell species produced following primary and secondary *C. jejuni* infections

**Figure 3:**
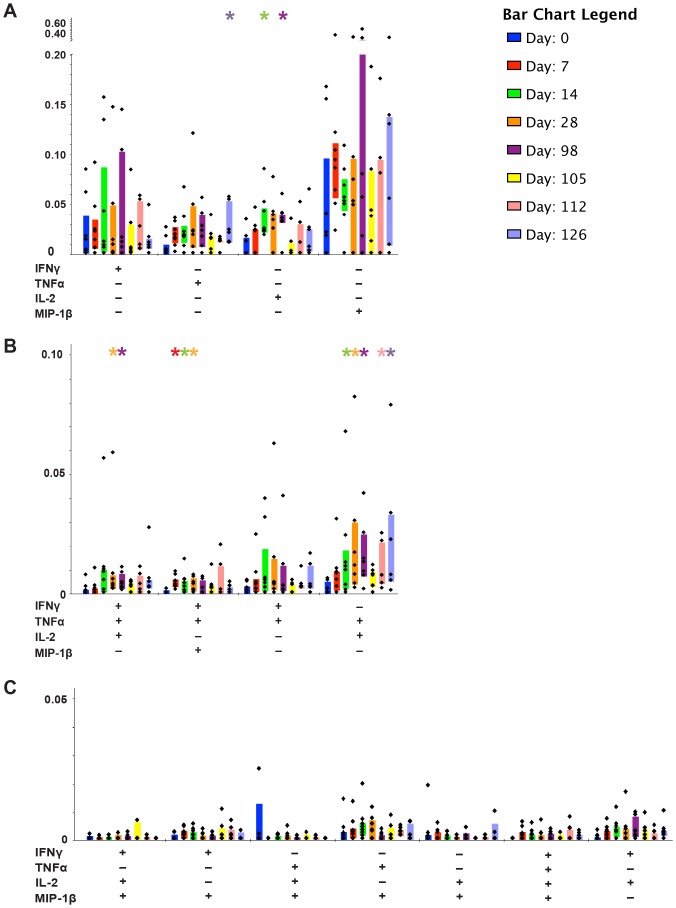
Monofunctional CD4^+^ T cells from naïve and veterans post *C. jejuni* exposure are dominant over multifunctional cells. The kinetics of T cell responses are shown, separating CD4^+^ phenotypes based on the combinations of cytokines they produced (designated by +/-) under the x-axis. X-axis shows the combinations of IFNγ, TNFα, IL-2, and MIP-1β producing cells. Each colored bar designates the interquartile range (IQR). Days evaluated include: D0, D7, D14, D28, D98, D105, D112, and D126. A) Monofunctional CD4^+^ phenotypes. B and C) Multifunctional CD4^+^ phenotypes relative to D0. Statistically significant days are relative to D0 (Wilcoxon ranked test) and P≤0.05 is designated by “*”.

Since we observed different T cell profiles between naïve and re-challenged subjects, we wanted to investigate the T cell functional diversity more closely. We used Boolean gating to evaluate the CD4^+^ T cell profiles; more specifically, to characterize whether responses were multifunctional or monofunctional, and to determine which cytokine patterns were prevalent. Although many of the cytokine-producing T cells were monofunctional CD4^+^IFNγ^+^ cells, statistically significant monofunctional populations included CD4^+^IL-2^+^ (D14 P<0.05), CD4^+^MIP-1β^+^ (D7 P<0.05), and CD4^+^TNFα^+^ (D7 P<0.05) (**Fig. 3A**). Monofunctional cytokine producing CD4^+^ T cells were more frequently produced over multifunctional cells for the overall study (P<0.0003). Low, but statistically significant percentages of multifunctional cells, were observed for the following T cell phenotypes and timepoints relative to pre-exposure: CD4^+^IFNγ^+^IL-2^+^TNFα^+^ (D14; P<0.05), CD4^+^IFNγ^+^MIP-1β^+^TNFα^+^ (D7, D14, D28, D105; P<0.01, 0.01, 0.05, and 0.05 respectively), CD4^+^IFNγ^+^IL-2^+^ (D14 and D98; P<0.05), and CD4^+^IL-2^+^TNFα^+^ (D28, D98, and D126; P<0.05) (**Fig. 3B**). CD4^+^IFNγTNFα^+^, CD4^+^IFNγ^+^IL-2^+^MIP-1β^+^, CD4^+^IFNγ^+^MIP-1β^+^, CD4^+^IFNγ^+^IL-2^+^MIP-1β^+^, CD4^+^IFNγ^+^MIP-1β^+^, CD4^+^IL-2^+^MIP-1β^+^TNFα^+^, CD4^+^MIP-1β^+^TNFα^+^, CD4^+^IL-2^+^MIP-1β^+^, CD4^+^IFNγ^+^IL-2^+^MIP-1β^+^TNFα^+^, and CD4^+^IFNγ^+^IL-2^+^ multifunctional cells were not significant at any timepoint (Fig. 3B and Fig. 3C).

### Protection after homologous rechallenge in a second challenge model

**Figure 4:**
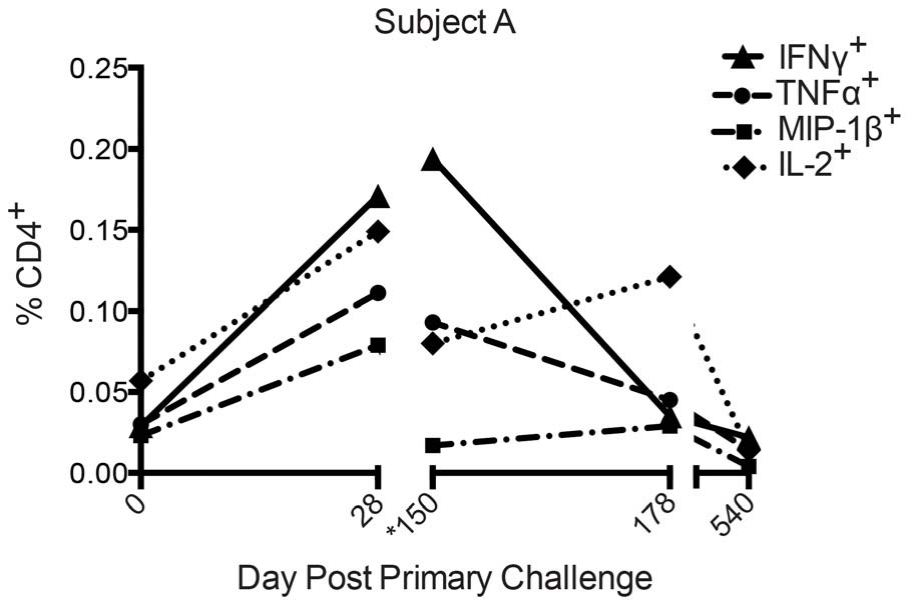
CD4^+^ T cells from protected Subject A produce proinflammatory cytokines following infection with live *C. jejuni*. PBMCs from pre-challenge (D0) and post-infection timeponts were stimulated with *C. jejuni* antigen in an *ex vivo* assay and assessed for CD4^+^ cytokine production by flow cytometry. Responses depicted represent the percentage of CD4^+^ T cells producing cytokine from CAg-stimulated PBMCs (CAg stimulation minus negative control). (*150 =  Day of homologous re-challenge.)

In a second Trial (Trial 2), using the same strain of *C. jejuni*, protection from clinical campylobacteriosis following homologous rechallenge was observed in one person. PBMCs isolated from Subject A one month after initial challenge (D28), were stimulated *ex vivo* using *C. jejuni* antigen and displayed increased numbers of IFNγ, TNFα, IL-2, and MIP-1β cytokine-producing CD4^+^ T cells relative to day of dosing, D0 (**Fig. 4**), suggesting a *C.jejuni* specific cellular immune response, similar to Trial 1. On the day of rechallenge (D150), TNFα, IL-2, and MIP-1β cytokine producing CD4^+^ T cells were back to baseline levels, however IFNγ CD4^+^ T cells were elevated relative to D0 (**Fig. 4**). Interestingly, there was a notable drop in IFNγ CD4^+^ T cells 28 days (D178) after re-challenge and further, all CD4^+^ T cells returned to baseline levels one year (D540) post re-challenge (**Fig. 4**). Subject A displayed similar monofunctional and multifunctional profiles to those from Trial 2 (data not shown).

### *C. jejuni* specific CD4^+^ IFNγ^+^ T cell phenotype is not distinct between protected and non-protected subjects

Re-challenge T cell profiles from Subjects B-I (Trial 2) were compared to that of Subject A, who showed no clinical illness upon re-challenge (**Fig. S3**). As shown in **Fig. S3B**, CD4^+^IFNγ^+^ and CD4^+^TNFα^+^ appeared elevated on the day of re-challenge (D150 for Subject A) relative to D0 (**Fig. S3A**), while CD4^+^IL-2^+^ and CD4^+^MIP-1β^+^ cells trended lower. Analysis of Subjects B-I showed that CD4^+^IFNγ^+^ cells had fallen close to baseline at re-challenge (D98) **(Fig. S3A and Fig. S3B**) relative to D0 with the exception of two Subjects (C and D); we found significant differences only in CD4^+^IL-2^+^ T cells between first and second challenges (D0 and D98, P <0.05); we did not observe differences for CD4^+^IFNγ^+^, CD4^+^TNFα^+^, or CD4^+^MIP-1β^+^ T cells.

To investigate the variable clinical outcomes, we compared T cell cytokine profiles in greater depth between the protected Subject A and unprotected subjects from Trial 2. We compared the dominant T cell functional populations: CD4^+^IFNγ^+^, at D0, D28, and day of re-challenge D98 or D150 (Trial 1 and Trial 2, respectively). Analysis of the median fluorescent intensity (MFI) of the CD4^+^IFNγ^+^ populations suggests that the Subject A (protected) CD4^+^IFNγ^+^ cells produced more IFNγ at D28 than CD4^+^ IFNγ^+^ cells from Subjects C and D at the same timepoint (**Fig. S4**). Only 2 of the 8 subjects reached IFNγ levels comparable to those exhibited by Subject A after primary infection.

## Discussion

To further investigate the previous observation that IFNγ production is associated with protection from campylobacteriosis, we performed the first kinetic evaluation of T cell cytokine responses following human *C. jejuni* infection [12]. We studied responses elicited by *C. jejuni* infection in a unique population of naïve human subjects (i.e. no prior history of campylobacteriosis), undergoing both primary and secondary infection (homologous re-challenge) with *C. jejuni* CG8421. We tested 8 subjects that experienced campylobacteriosis following primary and secondary infection to determine proinflammatory T cell profiles, and found that these subjects had an increase in pro-inflammatory T cells post primary infection but failed to boost upon secondary infection. Additionally, we tested a single subject that did not experience campylobacteriosis after re-infection and found that this subject had a similar pro-inflammatory T cell response post primary infection relative to the 8 subjects that experienced re-infection. Our data shows that following primary infection in all subjects, peripheral *C. jejuni*-specific CD4^+^ T cells produce pro-inflammatory cytokines, including IFNγ and TNFα, peaking 7-14 days post-infection. Similarly, the production of the chemokine, MIP-1β, peaks at day 7 post infection, whereas the peak IL-2 response was detected 14-28 days post-infection, suggesting a maturation of an effector cell response. Although the magnitude of cytokine responses varied between subjects, the timing and character of responses were similar. Lastly, we did not observe a clear pattern of cytokine responses by CD8^+^ populations, but this is likely because our assay was not optimized for CD8^+^ T cell stimulation due to the antigen used.

We anticipated that CD4^+^ cells would show a strong enhancement of cytokine production upon secondary exposure, particularly in the protected subject (Subject A), however this was not observed in any subject. While there were different dose amounts administered at rechallenge between Subject A and Subjects B-I, possibly explaining the lack of symptoms observed in Subject A, the percentage of CD4^+^ cells producing cytokines, including IFNγ, TNFα, and IL-2, appeared to fall or was unaltered in most subjects. Possible hypotheses that may explain these results include that i) memory T cells did not develop and thus were not present during secondary infection, as described in viral infections including HIV vaccine research [22]; ii) T cells may be unresponsive due to induction of tolerance or exhaustion [23]; iii) protective cells (Subject A) may reside in the gut and are not detectable in peripheral blood.

Our investigation of T cell heterogeneity showed that monofunctional CD4^+^ T cells were the primary phenotype observed after *C. jejuni* infection. More specifically, CD4^+^IFNγ^+^ T cells were the dominant cytokine-producing population following primary and secondary exposure, and did not correlate with clinical protection or severity of disease. As shown in **Fig. S4**, CD4^+^IFNγ^+^ T cells from the protected subject (Subject A) demonstrated a higher MFI than unprotected subjects with similar cytokine patterns, even though all subjects had similar percentages of CD4^+^IFNγ^+^ cells on the day of re-challenge. Increases in monofunctional CD4^+^ cells producing IL-2, TNFα, or MIP-1β were also detected following primary exposure but a discernable pattern post-secondary exposure was not detected.

These unexpected results, characterized in a young, healthy adult population without previous *C.jejuni* exposure, highlight the difficulty in understanding the complex human immune responses to bacterial infections at mucosal surfaces. Complicating the analysis, *C. jejuni* has marked strain-to-strain variability, including phase variation in capsular expression [24], [25]. Interestingly, our observation of recurrent campylobacteriosis following re-challenge with strain CG8421 was not observed in another challenge model using strain 81-176 (administered at a higher bacterial inoculum and displaying different capsular characteristics) [13]. The observation of a lack of a CD4^+^ cytokine response following secondary infection is consistent with the observation that IgA^+^ antibody secreting plasma cells also fail to boost after *C. jejuni* re-challenge [21]. It was previously noted that the serum IgG in these subjects boosted at Day 7 after initial challenge and displayed a drop after 3 months; interestingly, a boost after rechallenge was not observed [21]. Similarly a boost in serum IgA was noted at Day 7 post initial challenge which dropped almost to baseline after 3 months; a boost 7 days post rechallenge was observed but did not reach the titer observed after initial challenge before falling back to baseline [21]. In the context of the development of protective immunity, the detection of CD4^+^IFNγ^+^ monofunctional cells as the dominant CD4^+^ subset may be a marker of a poor quality immune response to primary infection, and may be less likely to lead to immunologic memory and protection [22]. Alternative hypotheses may explain our findings and will require further evaluation in future studies: Although not seen in our population, multiple cytokine-producing effector cells may still be necessary for the generation of a protective and/or memory response to *C. jejuni*. IFNγ, which has been associated with protection, may be produced by other cell types. Protection may also be found in the setting of high and sustained levels of IFNγ not seen in our subjects.

Further study of CD8^+^ T cells, which are important in clearing intracellular infections, may also provide valuable clues to the immune response to *C. jejuni*. Recent investigations have shown that *C. jejuni*-containing endosomes avoid fusion with lysosomes in epithelial cells, a feature reminiscent of intracellular *Mycobacterium tuberculosis* and *Salmonella* Typhimurium [26]–[28]. Additionally, the mechanisms of epithelial cell invasion and intracellular survival are being elucidated, further suggesting that CD8^+^ T cell responses may be important in clearing infection [29], [30]. While we investigated the CD8^+^ response and the chemokine MIP-1β, a chemokine important for recruitment of additional immune cells to sites on infection and produced by activated CD8^+^ and CD4^+^ cells, no clear patterns of CD8^+^ or MIP-1β responses were observed [31]–[33].

Further carefully constructed and detailed evaluations are needed to better understand the human immune response to *C. jejuni*. Although human challenge models are expensive and uncommon, future studies should address the role of different immune cell populations, including NK cells and Th17 cells, shown to be involved in a mouse model of *Campylobacter* infection [34]. Additionally, the importance of T cell priming by dendritic cells should be evaluated early post-infection, to characterize the initial development of *Campylobacter*-specific adaptive and memory responses and the expression of surface markers that regulate lymphocyte homing to the intestinal tract. Similarly, T cell phenotypes suggestive of the development of tolerance or T cell exhaustion following primary infection should be evaluated. Investigations into the acquisition of oral tolerance based on inoculum size will be particularly relevant. Given the global prevalence of this pathogen and its close associations with autoimmune post-infectious sequelae, further characterization of the human and mucosal immune responses to *C. jejuni* will be important to expand our ability to understand and control this clinically important pathogen.

## Supporting Information

Figure S1 **Flow cytometry analysis of T cells responding to CAg post** ***C. jejuni*** **infection.** The representative gating scheme displays the raw data analysis for determining the T cell response using an *ex vivo* assay. After sample collection, PBMCs were gated for singlets, lymphocytes, and live CD3^+^ T cells (first three histograms). The CD3^+^ T cells were subdivided into CD4^+^CD8^-^ (CD4^+^) and CD4^-^CD8^+^ (CD8^+^) populations. CD4^+^ (represented above) and CD8^+^ populations were then analyzed for cytokine production including IFNγ, TNFα, IL-2, and MIP-1β, for all conditions run (negative control, positive control, and CAg). Gates were based on FMO data. Signal over background (percent positive T cells) was based on the mean of the CAg results minus the negative control.
(TIF)

Figure S2 **CD8^+^ T cells from** ***C. jejuni*** **challenged subjects respond to CAg post infection.** Increases in CD8^+^IFNγ^+^ and CD8^+^MIP-1β^+^ were observed after primary infection time-points. Responses shown are the percentage of cytokine positive CD8^+^ T cells from CAg-stimulated PBMCs with the background percentage of cytokine-positive T cells in the negative control (PBS) subtracted. CD8^+^ T cells analyzed post-challenge: D7 (blue), D14 (red), and D28 (black). A) CD8^+^IFNγ^+^ T cells; and B) CD8^+^MIP-1β^+^ T cells. CD8^+^ T cells analyzed post re-challenge: D105 (blue), D112 (red), and D126 (black). C) CD8^+^IFNγ^+^ T cells; and D) CD8^+^MIP-1β^+^ T cells.
(TIF)

Figure S3 **Subjects C and D, not protected from re-infection, shared a similar cytokine profile to subject A.** Nine *C. jejuni* veterans, one from Trial 1 and eight from Trial 2, underwent homologous re-challenge (150 days) and 98 days post initial challenge, respectively). A) D0 pre-challenge CD4^+^ T cell profiles display low levels of cytokine production. B) On day of re-challenge, Subject A reflected CD4^+^ cells making IFNγ, TNFα, and IL-2. Subjects C and D displayed similar cytokine profiles to Subject A. PBMCs were not available for Subject B after re-infection.
(TIFF)

Figure S4 **IFNγ^+^ producing CD4^+^T from protected Subject A cells have a higher median fluorescence intensity that those produced by unprotected Trial 2 Subjects C and D.** Median Fluorescence Intensity (MFI) analysis for CD4^+^IFNγ^+^ T cells from timepoints including naïve D0, D28, and day of re-challenge (D150 and D98 for Trial 1 and Trial 2, respectively).
(TIF)
