# Supplementary figures and images for: Peripheral CD4+ T Cell Cytokine Responses Following Human Challenge and Re-Challenge with Campylobacter jejuni
Source: PLoS One. 2014 Nov 14;9(11):e112513. doi: 10.1371/journal.pone.0112513 (PMC4232357; doi:10.1371/journal.pone.0112513)

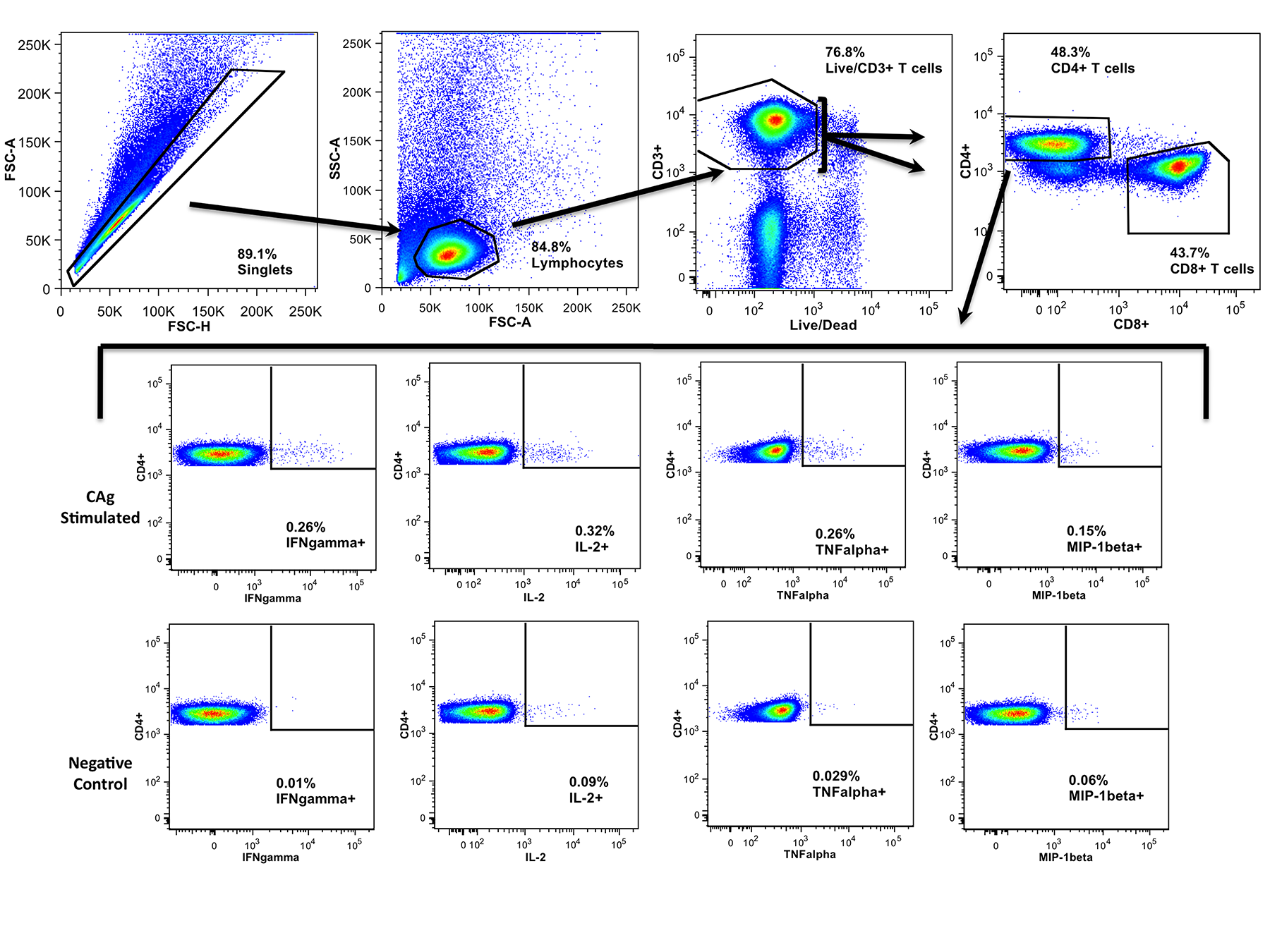

Supplement: Figure S1 — Flow cytometry analysis of T cells responding to CAg post C. jejuni infection. The representative gating scheme displays the raw data analysis for determining the T cell response using an ex vivo assay. After sample collection, PBMCs were gated for singlets, lymphocytes, and live CD3+ T cells (first three histograms). The CD3+ T cells were subdivided into CD4+CD8- (CD4+) and CD4-CD8+ (CD8+) populations. CD4+ (represented above) and CD8+ populations were then analyzed for cytokine production including IFNγ, TNFα, IL-2, and MIP-1β, for all conditions run (negative control, positive control, and CAg). Gates were based on FMO data. Signal over background (percent positive T cells) was based on the mean of the CAg results minus the negative control. (TIF) [file pone.0112513.s001.tif]

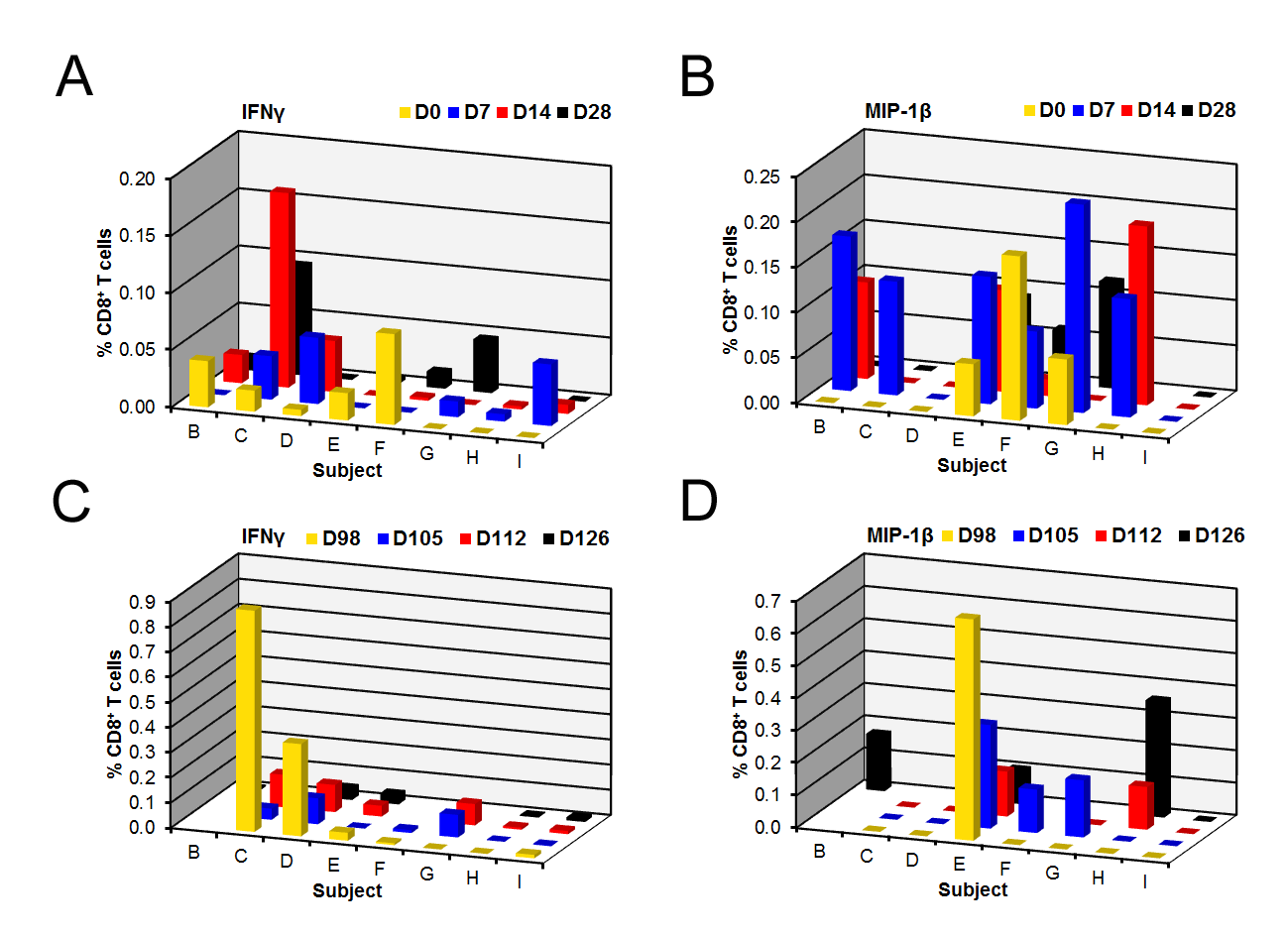

Supplement: Figure S2 — CD8+ T cells from C. jejuni challenged subjects respond to CAg post infection. Increases in CD8+IFNγ+ and CD8+MIP-1β+ were observed after primary infection time-points. Responses shown are the percentage of cytokine positive CD8+ T cells from CAg-stimulated PBMCs with the background percentage of cytokine-positive T cells in the negative control (PBS) subtracted. CD8+ T cells analyzed post-challenge: D7 (blue), D14 (red), and D28 (black). A) CD8+IFNγ+ T cells; and B) CD8+MIP-1β+ T cells. CD8+ T cells analyzed post re-challenge: D105 (blue), D112 (red), and D126 (black). C) CD8+IFNγ+ T cells; and D) CD8+MIP-1β+ T cells. (TIF) [file pone.0112513.s002.tif]

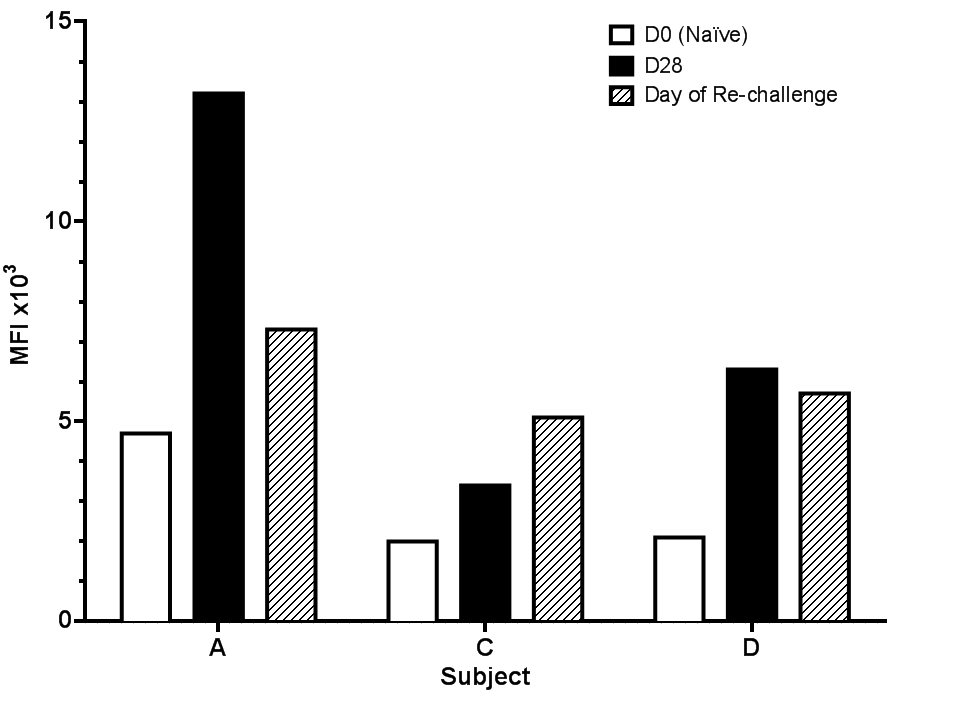

Supplement: Figure S4 — IFNγ+ producing CD4+T from protected Subject A cells have a higher median fluorescence intensity that those produced by unprotected Trial 2 Subjects C and D. Median Fluorescence Intensity (MFI) analysis for CD4+IFNγ+ T cells from timepoints including naïve D0, D28, and day of re-challenge (D150 and D98 for Trial 1 and Trial 2, respectively). (TIF) [file pone.0112513.s004.tif]
